# Assessing the appropriateness of the management of gastro-oesophageal reflux in Australian children: a population-based sample survey

**DOI:** 10.1038/s41598-021-87369-7

**Published:** 2021-04-08

**Authors:** Gaston Arnolda, Harriet Hiscock, David Moore, Glen Farrow, Peter D. Hibbert, Louise K. Wiles, Hseun P. Ting, Charlotte J. Molloy, Meagan Warwick, Jeffrey Braithwaite

**Affiliations:** 1 Centre for Healthcare Resilience and Implementation Science, Australian Institute of Health Innovation, Macquarie University, Level 6, 75 Talavera Road, Sydney, NSW 2109 Australia; 2 Population Health Theme, Murdoch Children’s Research Institute, Royal Children’s Hospital, Flemington Road, Parkville, VIC 3052 Australia; 3 Department of Paediatrics, The University of Melbourne, Melbourne, VIC 3010 Australia; 4 School of Population and Global Health, The University of Melbourne, Melbourne, VIC 3010 Australia; 5 Women’s and Children’s Hospital, 72 King William Road, North Adelaide, SA 5006 Australia; 6 Sydney Children’s Hospital Network, Prince of Wales Hospital, High Street, Randwick, NSW 2031 Australia; 7 Centre for Population Health Research, School of Health Sciences, University of South Australia, 101 Currie Street, Adelaide, SA 5001 Australia

**Keywords:** Translational research, Paediatric research

## Abstract

Gastro-oesophageal reflux (GOR) is a common physiological state in infants and young children, with gastro-oesophageal reflux disease (GORD) its pathological manifestation. Management of GOR/GORD requires elimination of possible underlying causes, parental reassurance, modification of feeding and symptom mitigation, monitoring, and referral to paediatricians if warning signs are present. Published clinical practice guidelines (CPGs) seek to support clinicians and improve management. This study aimed to measure the proportion of Australian GOR/GORD paediatric care that was in line with CPG recommendations. National and international CPGs for GOR/GORD were systematically identified and candidate indicators extracted; a Delphi process selected 32 indicators relevant to Australian paediatric care in 2012 and 2013. Medical records were identified in General Practices, the offices of general paediatricians, Emergency Departments and inpatient settings. Adherence to indicators was assessed by nine trained paediatric nurses undertaking retrospective medical record review. Medical records were reviewed in 115 healthcare sites; identifying 285 children, three-quarters aged < 1 year, who had 359 visits for management of GOR/GORD; 2250 eligible indicator assessments were performed. Estimated adherence rates are reported for 21 indicators with ≥ 25 assessments. Five indicators recommending differential diagnostic tests (e.g., urinalysis) for infants presenting with recurrent regurgitation and poor weight gain had ~ 10% adherence; conversely, avoidance of unrecommended tests (e.g., barium swallow and meal) was high (99.8% adherence: 95% CI 97.0–100). Avoidance of prescription of acid-suppression medication for *infants* at the first presentation was higher if they were healthy and thriving (86.9% adherence: 95% CI 86.0–96.8), intermediate if they had feeding refusal (73.1%: 95% CI 56.0–86.3) and lower if they presented with irritability and unexplained crying (58.8%: 95% CI 28.2–85.0). A guideline targeting Australian health professionals caring for infants and children with GOR/GORD is warranted, highlighting the importance of differential diagnostic testing and avoidance of acid-suppression medication in infants.

## Introduction

Gastro-oesophageal reflux (GOR) is the passage of gastric contents into the oesophagus, with or without regurgitation or vomiting. When GOR causes troublesome symptoms or complications, it can be diagnosed as gastro-oesophageal reflux disease (GORD)^1^. GOR is a normal and common physiological state in infants, peaking at 1–3 months of age and reducing in prevalence to 12 months^2,3^, and GORD a pathological state.

In the US, data from an administrative claims database estimated an incidence of diagnosis of GORD of 12.3% in children aged 0–1 years in 2005 (rising sharply from 3.4% in 2000), and about 1% in older children and adolescents^4^. A 2008 study of French children attending general practitioners (GPs) and paediatricians estimated GORD prevalence of 12.6% for children aged 0–1 years, 4.1% for children aged 2–11 years, and 7.6% for adolescents aged 12–17 years^5^. Estimates of the incidence or prevalence of GORD have not been published for Australian children.

Diagnosis of GORD in infants and children is difficult as symptoms cannot be reliably elicited, signs are non-specific, and simple and reliable diagnostic tests are not available. Management therefore requires the elimination of other possible causes and careful monitoring, symptom mitigation, and investigation or referral to specialists if warning signs are present^1^. Treatments include parental reassurance, modification of feeding and pharmaceutical treatment with acid-suppressing therapy^6^. There are significant concerns about inappropriate treatment, particularly with proton pump inhibitors (PPIs)^7^.

International clinical practice guidelines (CPGs) for the treatment of GOR/GORD were developed jointly by the European and North American Societies for Pediatric Gastroenterology, Hepatology and Nutrition (ESPGHAN/NASPHAN) in 2009^1^. A survey of European paediatricians in 2011–2012 found low compliance with the international CPGs; for example, 82% reported prescribing PPIs inappropriately^8^. After a randomly selected subset of participating paediatricians was trained in the guidelines, the over-prescription rate reduced to 29%^9^.

CareTrack Kids (CTK) assessed care of Australian children aged 0–15 years, in 2012 and 2013, to determine the proportion that received care in line with CPG recommendations for 17 common conditions: averaged across the targeted conditions, adherence was estimated to be 59.8% (95% CI 57.5–62.0), and at 52.8% (95% CI 45.7–59.9) for GOR/GORD overall^10^. This paper presents and discusses the CareTrack Kids results for GOR/GORD, at indicator level.

## Methods

The CTK methods have been described in detail elsewhere^10–12^. Key aspects, specifically relevant to GOR/GORD, are described below.

### Development of indicators

The RAND-UCLA method was modified and applied to develop indicators^13^. A systematic search for Australian and international CPGs for GOR/GORD, relevant for the years 2012–2013, yielded eight CPGs, from which 44 recommendations were extracted. Recommendations were screened and eight were excluded: seven due to indeterminate wording (e.g., actions ‘may’ or ‘could’ have been performed) and one guiding statement without a recommended action.

Proposed recommendations were ratified by experts over a two-stage multi-round modified Delphi process, which comprised an email-based three-round internal review and a two-round external review, using a custom-designed wiki^12^. Four clinicians were recruited for the internal (n = 3; two paediatricians and a GP) and external reviews (n = 1; a paediatrician)^14^. All reviewers completed their assignments independently to minimise group-think^15^.

In the internal review, reviewers recruited by the CTK research team scored each recommendation against three criteria (acceptability, feasibility and impact)^12^ and advised inclusion or exclusion. For the external review, experts were recruited through advertising to the professional colleges; volunteers registered to an online wiki and self-nominated for CTK conditions based on their experience^14^. The external reviewer applied the same scoring criteria as internal reviewers and, in addition, used a nine-point Likert scale to score each recommendation as representative of appropriate care delivered to children during 2012 and 2013^12,13^. The clinical lead for each condition commented on reviewers’ responses, and made final decisions regarding the inclusion of the recommendations. A total of 15 final recommendations were ratified by experts and formatted into 32 medical record audit indicator questions. All indicator questions are shown, with additional details, in eTable 1, Appendix 1.

### Sample size, sampling process and data collection

CTK targeted 400 medical records for GOR/GORD and 6000 medical records for 16 other conditions. If any of the 6400 medical records targeted and sampled contained an occasion of care for GOR/GORD, the visit was assessed. Detail on the general sampling methods are provided in the main paper^10^; additional details specific to GOR/GORD can be found in in Appendix 2. Briefly, four healthcare settings were sampled: hospital inpatients and Emergency Department (ED) presentations, and consultations with GPs and private-practice paediatricians, in selected health districts in the Australian states of Queensland, New South Wales and South Australia, for children aged ≤ 15 years receiving care in 2012 and 2013. The overall recruitment rate was 92% for hospitals and estimated to be 25% for paediatricians and 24% for GP (Appendix 2). Data were collected by nine experienced paediatric nurses, trained to assess eligibility for indicator assessment and compliance with CPGs.

### Analysis

At indicator level, estimates of compliance were measured as the percentage of eligible indicators which scored as adherent. Weights were constructed to adjust of oversampling of some states, settings and sites^10^. The weighted data were analysed in SAS v9.4 (SAS Institute, Cary NC, USA), using the SURVEYFREQ procedure. Variance was estimated by Taylor series linearization. State and healthcare setting were specified as strata, with the primary sampling unit (health district) specified as the clustering unit. Exact 95% CIs were generated using the modified Clopper–Pearson method. Results were suppressed if there were < 25 eligible visits.

### Ethical considerations

Primary ethics approval was received from the New South Wales Sydney Children’s Hospital Network Ethics Committee (HREC/14/SCHN/113), the Queensland Government Human Research Ethics Committee (HREC/14/QRCH/91), the South Australian Women’s and Children’s Health Network Ethics Committee (HREC/14/WCHN/68) and the Royal Australian College of General Practitioners Ethics Committee (NREEC 14-008), and site-specific approvals from 34 hospitals. Informed consent was not sought from patients; Australian human research ethics committees can waive requirements for patient consent for external access to medical records if the study entails minimal risk to facilities, clinicians, and patients, and all four abovementioned committees provided this waiver. Informed written consent was received from all participating hospitals and paediatrician and general practitioner practices. Participating health care providers were protected from litigation by gaining statutory immunity for this study as a quality assurance activity from the Federal Minister for Health under Part VC of the Australian Health Insurance Act 1973. All methods were carried out in accordance with relevant guidelines and regulations.

## Results

Details of the 285 children with one or more eligible assessments of CPG adherence for GOR/GORD are provided in Table 1. Seventy-nine percent of the children in the CTK sample were aged under one year, with slightly more males (55%) than females. Each child had between one and four occasions of care for GOR/GORD which were eligible for assessment (median = 1).

**Table 1:** Characteristics of the eligible children, 2012–2013.

Characteristic	Children in the CTK study N = 285
**Age**^**a**^**—no. (%)**	
< 1 year	225 (78.9)
1–2 years	19 (6.7)
3–4 years	2 (0.7)
5–11 years	19 (6.7)
12–15 years	20 (7.0)
**Male—no. (%)**	156 (54.7)

^a^The child’s age was calculated as the age at visit where there was only one, or the midpoint of the child’s age at her first and last GORD visit, where there was more than one.

**Figure 1:**
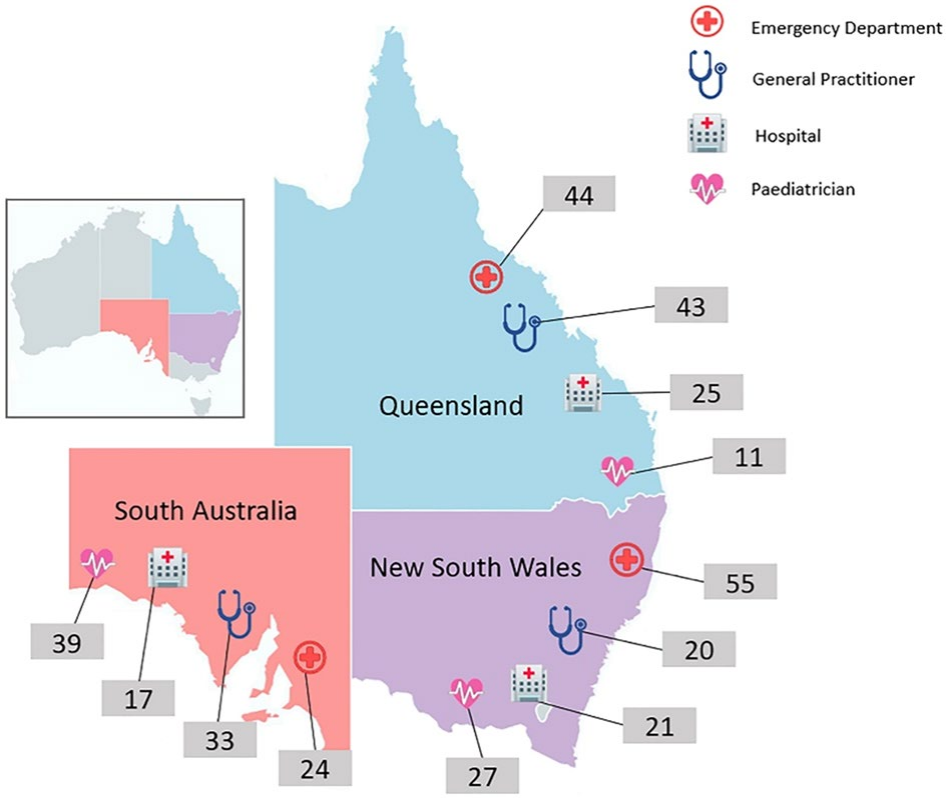
GORD assessments by state and health care provider type. Total number of visits to Emergency Departments = 123; total number of visits to General Practitioners = 96; total number of admissions to hospital = 63; total number of visits to Paediatricians = 77. Total number of GORD assessments in: New South Wales = 123; Queensland = 123; and South Australia = 113. Total number of visits assessed for care of GORD in sampling frame = 359. [Map outline from https://mapchart.net/, Creative Commons Attribution-ShareAlike 4.0 International License, with data added by the authors in Microsoft PowerPoint for Microsoft 365 MSO Version 2102].

Of 12,480 possible indicator assessments, 3648 (29.2%) were filtered out by age and healthcare setting restrictions and 6582 (52.7%) were designated as not applicable by surveyors. The field team conducted 2250 eligible indicator assessments during 359 visits, at a median of 6 indicators per visit. Eligible GORD visits were assessed in 32 GP and 14 SP practices, 33 hospital EDs and 26 hospital inpatient sites. The distribution of visits by state and provider is summarised in Fig. 1.

### Adherence

Estimated adherence for 21 of the 32 indicators is shown in Table 2; 11 indicators are not reported because they had < 25 visits. For the 21 indicators where adherence was assessed, compliance ranged from 8.5% for indicator GORD10 (“Infants […] with recurrent regurgitation and poor weight gain despite adequate energy intake had their blood urea nitrogen assessed”) to 99.8% for GORD14 (“Infants/children who presented with uncomplicated recurrent regurgitation did not have a barium swallow and meal”), the latter being the percentage avoiding an unrecommended diagnostic test. The interquartile range for adherence in the 21 indicators reported was 11.6% to 73.1%.

**Table 2:** Appropriateness of care, by clinical indicator.

Indicator ID	Indicator description	No. of eligible children	No. of eligible surveys	Percentage appropriateness per indicator (95% CI)
GORD01	Infants/children who presented with regurgitation had their weight and height (growth chart) documented	234	292	44.4 (33.7, 55.5)
GORD02	Infants/children who presented with regurgitation had their allergies (skin rash/urticaria/eczema/diarrhoea/perineal/perianal excoriation), food and milk intolerances (cow's milk) documented	233	293	37.3 (19.3, 58.3)
GORD03	Infants/children aged ≥ 6 years who presented with regurgitation had their history of regurgitation/vomiting, cough, epigastric pain/heartburn documented	23	24	Insufficient data
GORD04	Infants/children who presented with a history of food refusal OR regurgitation/vomiting, had their weight and height (growth chart) recorded	230	293	67.3 (32.4, 91.9)
GORD05	Infants/children who presented with a history of food refusal OR regurgitation/vomiting, received a urine MC&S	232	291	11.6 (3.5, 26.4)
GORD06	Infants aged less than 12 months with recurrent regurgitation and poor weight gain despite adequate energy intake have their diet history assessed	22	32	87.6 (59.2, 98.8)
GORD07	Infants aged less than 12 months with recurrent regurgitation and poor weight gain despite adequate energy intake received a urinalysis	20	29	10.6 (2.3, 27.7)
GORD08	Infants aged less than 12 months with recurrent regurgitation and poor weight gain despite adequate energy intake received a complete blood count	20	29	9.4 (1.8, 26.2)
GORD09	Infants aged less than 12 months with recurrent regurgitation and poor weight gain despite adequate energy intake had their serum electrolytes assessed	20	29	9.4 (1.8, 26.2)
GORD10	Infants aged less than 12 months with recurrent regurgitation and poor weight gain despite adequate energy intake had their blood urea nitrogen assessed	20	29	8.5 (1.4, 25.0)
GORD11	Infants aged less than 12 months with recurrent regurgitation and poor weight gain despite adequate energy intake had their serum creatinine assessed	20	29	8.8 (1.5, 25.4)
GORD12	Infants who had uncomplicated recurrent regurgitation "happy spitters" had their feeding and feeding practices reviewed	74	87	48.4 (6.0, 92.7)
GORD13	Infants who had uncomplicated recurrent regurgitation "happy spitters" were provided with parental reassurance and education	75	88	55.1 (7.8, 95.7)
GORD14	Infants/children who presented with uncomplicated recurrent regurgitation did not have a barium swallow and meal	122	147	99.8 (97.0, 100)
GORD15	Children aged greater than 18 months who presented with dysphagia or odynophagia were referred to a paediatric gastroenterologist	5	6	Insufficient data
GORD16	Children aged greater than 18 months who presented with dysphagia or odynophagia received a barium swallow	5	6	Insufficient data
GORD17	Infants with reflux who were healthy and thriving and presented with irritability or unexplained crying were not prescribed acid suppression medication at the first presentation	80	92	58.8 (28.2, 85.0)
GORD18	Infants with reflux who were healthy and thriving and presented with feeding refusal were not prescribed acid suppression medication at the first presentation	31	37	73.1 (56.0, 86.3)
GORD19	Infants with reflux who were healthy and thriving and presented with frequent regurgitation were not prescribed acid suppression medication at the first presentation	111	125	86.9 (68.0, 96.8)
GORD20	Children with Barrett's Oesophagus had multiple biopsies obtained at time of endoscopy	2	2	Insufficient data
GORD21	Children with Barrett's Oesophagus were prescribed acid suppression	2	2	Insufficient data
GORD22	Older children/adolescents who presented with heartburn were assessed for lifestyle factors (diet, alcohol, weight, sleeping position, smoking)	13	13	Insufficient data
GORD23	Older children/adolescents who presented with heartburn were prescribed a PPI for 4 weeks	11	11	Insufficient data
GORD24	Older children/adolescents who presented with heartburn, had been prescribed and used a PPI for 4 weeks, and their symptoms had resolved/improved were reviewed by their GP and had their PPI continued for 3 months	4	4	Insufficient data
GORD25	Older children/adolescents who presented with heartburn, had been prescribed and used a PPI for 4 weeks, and they had recurrent/persistent symptoms were reviewed by their GP and referred to a gastroenterologist	1	1	Insufficient data
GORD26	Infants/children (aged less than 18 months) with reflux oesophagitis had their family lifestyle factors recorded (diet, alcohol, weight, sleeping position, smoking)	44	55	53.1 (8.6, 93.9)
GORD27	Infants/children (aged less than 18 months) with reflux oesophagitis had their symptoms reassessed at each review	25	29	80.7 (28.1, 99.6)
GORD28	Infants/children who had the presence of warning signs (see definition^a^) were referred to a paediatric gastroenterologist	42	57	55.5 (9.7, 94.7)
GORD29	Infants/children who had difficulty swallowing or a history of obstruction were referred to a paediatric gastroenterologist	14	16	Insufficient data
GORD30	Infants/children who had weight loss/anorexia/poor feeding were referred to a paediatric gastroenterologist	42	57	85.9 (51.0, 99.1)
GORD31	Infants/children whose symptoms persisted during and after PPI therapy were referred to a paediatric gastroenterologist	37	45	49.1 (18.3, 80.5)
GORD32	Infants/children with uncomplicated recurrent regurgitation who presented with projectile vomiting OR haematemesis OR bile-stained vomiting, were immediately referred to a hospital emergency department	0	0	Insufficient data

*MC&S* Microscopy, Culture and Sensitivities, *PPI* Proton-Pump Inhibitor.

^a^Includes: Bilious vomiting; Gastrointestinal bleeding; Hematemesis; Hematochezia; Consistently forceful vomiting; Onset of vomiting after 6 months of life; Failure to thrive; Diarrhea; Constipation; Fever; Lethargy; Hepatosplenomegaly; Bulging fontanelle; Macro/microcephaly; Seizures; Abdominal tenderness or distension; Documented or suspected genetic/metabolic syndrome.

There was poor recording of weight and height for infants and children who presented with regurgitation (44.4%; 95% CI 33.7–55.5; GORD01), but a higher estimated rate (albeit with wider confidence intervals) if they presented with a *history* of food refusal or regurgitation (67.3%; 95% CI 32.4–91.9; GORD04). There was poor recording of allergies, food and milk intolerances (37.3%; 95% CI 19.3–58.3; GORD02).

Infants presenting with recurrent regurgitation and poor weight gain despite adequate energy intake (n = 29–32 visits) appeared poorly served, overall. There was good recording of diet history (87.6%; 95% CI 59.2–91.9; GORD06), but poor compliance with guidelines for diagnostic investigations including urinalysis (10.6%; 95% CI 2.3–27.7; GORD07), complete blood count and assessment of serum electrolytes (GORD08 and GORD09; each 9.4%; 95% CI 1.8–26.2), blood urea nitrogen (8.5%; 95% CI 1.4–25.0; GORD10) and serum creatinine (8.8%; 95% CI 1.5–25.4; GORD11). Poor compliance with diagnostic testing is also seen in the performance of urine microscopy, culture and sensitivities (11.6%; 95% CI 3.5–26.4; GORD05).

Avoiding prescription of acid suppression medications on the first presentation of infants with reflux who were healthy and thriving varied according to other characteristics of the presentation: compliance was highest for infants presenting with frequent regurgitation (86.9%; 95% CI 68.0–96.8; GORD19), intermediate for infants presenting with feeding refusal (73.1%; 95% CI 56.0–86.3; GORD18) and lowest, but with wide confidence intervals, for infants presenting with irritability or unexplained crying (58.8%; 95% CI 28.2–85.0; GORD17).

Three indicators which measure compliance with guidelines to refer children to a paediatric gastroenterologist also had variable results. Estimated compliance was high for infants and children who had weight loss or anorexia or poor feeding (85.9%; 95% CI 51.0–99.1; GORD30) and lower, with wider confidence intervals, for those with selected warning signs (55.5%; 95% CI 9.4–94.7; GORD28) and for those whose symptoms persisted during and after PPI therapy (49.1%; 95% CI 18.3–80.5; GORD31).

## Discussion

A wide range of estimated adherence to guidelines was found for management of GOR/GORD in children. Given the importance of failure to thrive as a warning sign^1^, it was disappointing that infants and children presenting with regurgitation did not routinely have weight and height recorded. The low level of routine documentation of allergies and food intolerance is similarly concerning.

The ESPGHAN/NASPHAN guidelines conclude that history and physical examination are sufficient for a diagnosis of GORD in older children and adolescents, but not infants and younger children. Compliance was consistently around 10% for a series of five differential diagnostic tests for infants presenting with recurrent regurgitation with poor weight gain (urinalysis, complete blood count, serum electrolytes, blood urea nitrogen, and serum creatinine), and separately for urine microscopy, culture and sensitivities for infants and children presenting with a history of food refusal or recurrent regurgitation. However, the five diagnostic tests for infants with recurrent regurgitation and poor weight gain are only recommended by the 2009 ESPGHAN/NASPHAN guideline^1^, while the 2018 ESPGHAN/NASPHAN guideline notes that unspecified tailored tests should be undertaken to address differential diagnosis of alarm signs^16^; an Australian guideline notes the role of investigations for differential diagnosis, but does not recommend specific tests^6^.

There was a high rate of referral to a paediatric gastroenterologist (86%) for infants and children with anorexia, weight loss or poor feeding, but poorer rates of referral for children with warning signs (56%; see note to Table 2 for full listing of signs) and for children whose symptoms persisted during and after PPI therapy (49%). By contrast, a 2011 survey of Australian GPs found that 81% of respondents stated that they refer to a specialist following an ineffective medication trial for GORD^17^. The difference may reflect a bias in self-reporting, or a lack of affordable access to specialists—in a recent Australian survey, 27% of GPs noted lack of access to specialists and 22% noted patient financial status as issues influencing their management of infant GOR/GORD^17^.

Among indicators concerned with *overuse* of unrecommended treatments, compliance was variable for the prescription of PPIs at the first presentation of a healthy thriving infant, depending on the presentation: 87% for infants with frequent regurgitation; 73% for infants with feeding refusal; and 59% for irritable infants or with unexplained crying, but with wide confidence intervals. A survey of general paediatricians in 11 European countries found that 63% appropriately avoided prescribing PPIs for infants with recurrent regurgitation and 55% avoided prescribed PPIs for unexplained crying or distress; a survey of Italian paediatricians found rates of appropriate avoidance of PPI prescription of 62% and 44%, respectively; and a 2002–2004 survey of US Family Practitioners found self-reported rates of avoidance of empirical trials of acid suppression of 70% and 38%, respectively. It is important to note that the European, Italian and US estimates are based on clinicians’ responses to case scenarios, while the CTK estimates focus on the first presentation of a child, excluding subsequent consultations. That said, the estimated performance of Australian clinicians appears higher in relation to children presenting with uncomplicated recurrent regurgitation, but broadly similar in relation to infants with unexplained distress. The management of these infants is of concern, as a recent meta-analysis of five randomised controlled trials on the use of PPIs on crying and irritable infants found no evidence of benefit^18^; only one trial reported adverse events, and this found a statistically significant increase in serious adverse events, associated with a higher rate of lower respiratory tract infections^19^.

Australian data on the use of prescription medicines as first line therapy in infants with GORD is limited. A survey of GPs found that 16% would ‘usually’ use prescription medicines as first line therapy for GORD, with an additional 56% doing so ‘occasionally’; details were not reported by type of medicine or clinical presentation, but it is notable that a majority of GPs consider PPIs to be highly effective^17^. Studies in the US^4^ and in Belgium^20^ have found marked increases in the use of PPIs for paediatric patients, with concerns about misuse, particularly in infants and younger children.

The authors are aware of one interventional study that sought to improve compliance with CPGs for management of GOR/GORD in children. Baseline data were taken from a 2011–2012 random survey of general paediatricians in 11 European countries, with respondents (42%) providing information about their diagnosis and treatment for 12 clinical scenarios^8^, to assess adherence to the ESPGHAN/NASPHAN guideline^1^. A follow-up study trained the first 100 respondents, from a randomly selected listing, who agreed to participate in an intervention and to record details on the management of each child managed for GOR/GORD in the three months following^9^. Two interventions were assessed: a 30-min podcast and set of slides vs a written synopsis of the guidelines; follow-up data was aggregated as no differences were noted between the intervention groups. Results of the data audit for the 100 participants post-intervention were compared to their baseline case scenario responses. The impact of intervention on self-reported practice was marked: 37.1% of paediatricians stated that they would use a PPI for an infant with recurrent regurgitation in response to the pre-intervention case scenario but only 4.5% recorded use of a PPI for these infants in the post-training audit of practice; the corresponding rates were 45.2% pre- and 3.7% post-intervention for PPI use in infants with unexplained crying or distressed behaviour. Whether this pattern would continue in routine practice, unaccompanied by audit, is unknown.

Despite the differential outcome measurement pre- and post-intervention and the possibility of response bias in the post-intervention period, the results suggest that substantial changes in clinical behaviour of general paediatricians might be achieved with minimal intervention. In Australia, an intensive clinical audit and feedback program to improve management of GORD in adults attending GPs reported some successes in improving compliance with evidence-based guidelines^21^, but the changes achieved were marginal.

A 2011 survey of Australian GPs about the management of GOR/GORD in infants found that 37% of respondents were concerned about the lack of guidelines and education relevant to infants^17^. GORD is *discussed* in a number of current guidelines including: the National Health and Medical Research Council guideline on infant feeding^22^; consensus panel guidelines for cough in children and adults^23^; and on use of infant formulas to treat cows’ milk protein allergy^24^. Management guidance specifically addressing paediatric presentations with GOR/GORD have also been published for Australian GPs^6^, and the literature has also been recently reviewed for health professionals with an interest in breastfeeding^25^. Internationally, comprehensive guidelines that were published jointly by ESPGHAN/NASPHAN in 2009 were used in the current study^1^, updated^26^, and supported by the American Academy of Pediatrics^27^; a detailed literature review and separate guidance was also published by the UK National Institute for Clinical Excellence (NICE)^28^, and the ESPGHAN/NASPHAN guideline was updated in 2018^16^. This reflects growing recognition of the need to provide health professionals with guidance on the management of GOR/GORD in infants and children, to reduce unwarranted clinical variation and improve management.

The CTK study has numerous strengths and weaknesses which are described in additional detail elsewhere^10^ and summarised briefly here. The data are drawn from a large survey in three States covering 60% of Australia’s paediatric population. For logistical reasons, coverage was limited to larger hospitals providing ~ 40% of all inpatient and ED care. While hospitals had excellent participation rates, the participation rates of GPs and specialist providers is estimated to be ~ 25%; it is plausible that self-selection has led to over-estimation of compliance. Within healthcare sites, random record selection was externally controlled in both hospital and GP settings, but random record selection could not be standardised in paediatricians consulting rooms, with unknown impacts on estimated compliance. The results of 11 indicators had < 25 responses and were thus too unreliable to publish; the results of several other indicators had wide confidence intervals. Finally, this study assesses documented practice, and it is plausible that this differs from actual practice; in primary care, it has been estimated lack of documentation can lead to underestimation of compliance by around 10 percentage points^29^.

Gaining ethical and administrative clearance to audit over 6000 thousand medical records in over 100 health facilities, undertaking the audits and reporting on each of the audited clinical conditions has led to a substantial passing of time from the period audited (2012–2013) to the time of publication. Clearly, clinical behaviour may have changed in the interim. Moreover, two international clinical guidelines on paediatric GOR/GORD have been published since 2013, the NICE guideline in 2015^28^ and the 2018 update of the ESPGHAN/NASPHAN guideline^16^. While these guidelines have not substantively altered the principles underlying the indicators assessed in 2012–2013, some details have changed. For example, the 2009 ESPGHAN/NASPHAN recommendations for five specific tests for regurgitating infants with poor weight gain has been replaced in the 2018 guideline by advice to undertake unspecified tests appropriate to address differential diagnosis for ‘alarm signs’. It is noteworthy, however, that the 2018 guidance is effectively unchanged from 2009 in recommending the avoidance of PPIs for infants who are healthy and thriving, even if distressed, while acknowledging that the parents of distressed infants often place clinicians under intense pressure to provide a ‘solution’. The overuse of PPIs in infants has been noted as an important example of irrational prescribing in children^30^, for which solutions must be found.

The results suggest that children and infants with GOR/GORD, and their families, could be better served. The health system needs to provide detailed guidance for clinicians involved in the care of these children, providing standards against which they can benchmark and monitor their performance. Such guidance needs to cover simple assessment, such as charting of weight and height, judicious diagnostic testing for differential and positive diagnosis, and avoidance of unjustified pharmaceutical interventions such as use of PPIs in healthy infants who present with irritability and unexplained crying. In addition to interventions targeting clinician behaviour, the needs and beliefs of parents of children with GOR/GORD, and with unexplained crying, need to be understood as a basis for designing interventions targeting parents. This understanding also open the possibility of targeting the clinician-parent interaction through innovative interventions such as shared decision-making programs, which have been demonstrated to be effective in reducing the unwarranted prescription of antibiotics^31^.

## Conclusion

The study shows poor overall compliance with CPGs in the management of infants and children presenting with GOR/GORD, with some recommended actions showing very poor compliance and others showing extremely high compliance. The results suggest that a guideline targeting Australian health professionals caring for infants and children with GOR/GORD is warranted, with multi-faceted interventions to promulgate guidelines and increase sustained compliance, supported by systems of ongoing monitoring and feedback to individual clinicians.

## Supplementary Information

Supplementary Appendix 1.

Supplementary Appendix 2.

## Data Availability

The datasets used and/or analysed during the current study are available from the corresponding author on reasonable request.

## Abbreviations

CPGs: Clinical practice guidelines
ED: Emergency department
ESPGHAN/NASPHAN: European and North American Societies for Pediatric Gastroenterology, Hepatology and Nutrition
GOR: Gastro-oesophageal reflux
GORD: Gastro-oesophageal reflux disease
GP: General practitioner
NICE: UK National Institute for Clinical Excellence
PPIs: Proton pump inhibitors
