# Supplementary material for: Assessing the appropriateness of the management of gastro-oesophageal reflux in Australian children: a population-based sample survey
Source: Sci Rep. 2021 Apr 8;11:7744. doi: 10.1038/s41598-021-87369-7 (PMC8032666; doi:10.1038/s41598-021-87369-7)
Supplement: Supplementary file 1 — Supplementary Appendix 1. [file 41598_2021_87369_MOESM1_ESM.docx]

**Assessing the appropriateness of the management of Gastro-Oesophageal Reflux in Australian children: a population-based sample survey**

**Authors:**

Gaston Arnolda, PhD^1*^, Harriet Hiscock, MD^2,3,4^, David Moore, MBBS^5^, Glen Farrow, MBA^6^, Peter D. Hibbert, Grad Dip. Econ^1,7^, Louise K. Wiles, PhD^1,7^, Hseun P. Ting, MSc^1^, Charlotte J. Molloy, BBehSc^1,7^, Meagan Warwick, MSc^1^, and Jeffrey Braithwaite, PhD^1^, *on behalf of the CareTrack Kids Investigative Team*

^1^ Australian Institute of Health Innovation, Macquarie University, Level 6, 75 Talavera Road, Sydney, NSW 2109

^2^ Population Health Theme, Murdoch Children’s Research Institute, Royal Children’s Hospital, Flemington Road, Parkville, VIC 3052

^3^ Department of Paediatrics, The University of Melbourne, Melbourne, VIC 3010

^4^ School of Population and Global Health, The University of Melbourne, Melbourne, VIC 3010

^5^ Women’s and Children’s Hospital, North Adelaide, 72 King William Road, SA 5006

^6^ Sydney Children's Hospital Network, Prince of Wales Hospital, High Street, Randwick, NSW 2031

^7^ Centre for Population Health Research, School of Health Sciences, University of South Australia, 101 Currie Street, Adelaide, SA 5001

***Corresponding author:**

**Dr Gaston Arnolda**

Centre for Healthcare Resilience and Implementation Science

Australian Institute of Health Innovation, Macquarie University

Level 6, 75 Talavera Road, Macquarie University | NSW | 2109

**Email: gaston.arnolda@mq.edu.au**; Phone +61 2 9850 2411; Fax +61 2 9850 2499

**Appendix 1: Additional characteristics of indicators and number of sites where indicator measured**

**eTable 1: Characteristic of GOR/GORD clinical indicators**

|  | | | **No. of Sites** | | | |  | | |
| --- | --- | --- | --- | --- | --- | --- | --- | --- | --- |
| **Indicator ID** | **Indicator Description** | **Age Inclusion Criteria** | **GP** | **P** | **ED** | **INPT** | **Strength of Recommendation^#^** | **Care Type** | **Quality Type*** |
| GORD01 | Infants/children who presented with regurgitation had their weight and height (growth chart) documented. | 0 - 15 years | 26 | 14 | 32 | 26 | Consensus-based recommendation | Diagnosis | Underuse |
| GORD02 | Infants/children who presented with regurgitation had their allergies (skin rash/urticaria/eczema/diarrhoea/perineal/perianal excoriation), food and milk intolerances (cow's milk) documented. | 0 - 15 years | 26 | 14 | 32 | 26 | Consensus-based recommendation | Diagnosis | Underuse |
| GORD03 | Infants/children aged ≥ 6 years who presented with regurgitation had their history of regurgitation/vomiting, cough, epigastric pain/heartburn documented. | 6 - 15 years | 8 | 2 | 6 | 3 | Consensus-based recommendation | Diagnosis | Underuse |
| GORD04 | Infants/children who presented with a history of food refusal OR regurgitation/vomiting, had their weight and height (growth chart) recorded. | 0 - 15 years | 25 | 14 | 31 | 25 | Consensus-based recommendation | Diagnosis | Underuse |
| GORD05 | Infants/children who presented with a history of food refusal OR regurgitation/vomiting, received a urine MC&S. | 0 - 15 years | 26 | 14 | 31 | 25 | Consensus-based recommendation | Diagnosis | Underuse |
| GORD06 | Infants aged less than 12 months with recurrent regurgitation and poor weight gain despite adequate energy intake have their diet history assessed. | 0 - 11 months | 4 | 4 | 9 | 6 | Grade D | Diagnosis | Underuse |
| GORD07 | Infants aged less than 12 months with recurrent regurgitation and poor weight gain despite adequate energy intake received a urinalysis. | 0 - 11 months | 4 | 3 | 8 | 6 | Grade D | Diagnosis | Underuse |
| GORD08 | Infants aged less than 12 months with recurrent regurgitation and poor weight gain despite adequate energy intake received a complete blood count. | 0 - 11 months | 4 | 3 | 8 | 6 | Grade D | Diagnosis | Underuse |
| GORD09 | Infants aged less than 12 months with recurrent regurgitation and poor weight gain despite adequate energy intake had their serum electrolytes assessed. | 0 - 11 months | 4 | 3 | 8 | 6 | Grade D | Diagnosis | Underuse |
| GORD10 | Infants aged less than 12 months with recurrent regurgitation and poor weight gain despite adequate energy intake had their blood urea nitrogen assessed. | 0 - 11 months | 4 | 3 | 8 | 6 | Grade D | Diagnosis | Underuse |
| GORD11 | Infants aged less than 12 months with recurrent regurgitation and poor weight gain despite adequate energy intake had their serum creatinine assessed. | 0 - 11 months | 4 | 3 | 8 | 6 | Grade D | Diagnosis | Underuse |
| GORD12 | Infants who had uncomplicated recurrent regurgitation "happy spitters" had their feeding and feeding practices reviewed. | 0 - 11 months | 9 | 6 | 20 | 14 | Grade C | Treatment | Underuse |
| GORD13 | Infants who had uncomplicated recurrent regurgitation "happy spitters" were provided with parental reassurance and education. | 0 - 11 months | 9 | 6 | 21 | 14 | Grade C | Treatment | Underuse |
| GORD14 | Infants/children who presented with uncomplicated recurrent regurgitation did not have a barium swallow and meal. | 0 - 15 years | 22 | 11 | 25 | 19 | Consensus-based recommendation | Treatment | Overuse |
| GORD15 | Children aged greater than 18 months who presented with dysphagia or odynophagia were referred to a paediatric gastroenterologist. | 18 months - 15 years | 2 | 0 | 3 | 0 | Consensus-based recommendation | Treatment | Underuse |
| GORD16 | Children aged greater than 18 months who presented with dysphagia or odynophagia received a barium swallow. | 18 months - 15 years | 2 | 0 | 3 | 0 | Grade D | Treatment | Underuse |
| GORD17 | Infants with reflux who were healthy and thriving and presented with irritability or unexplained crying were not prescribed acid suppression medication at the first presentation. | 0 - 11 months | 12 | 8 | 21 | 12 | Consensus-based recommendation | Treatment | Overuse |
| GORD18 | Infants with reflux who were healthy and thriving and presented with feeding refusal were not prescribed acid suppression medication at the first presentation. | 0 - 11 months | 2 | 2 | 15 | 7 | Consensus-based recommendation | Treatment | Overuse |
| GORD19 | Infants with reflux who were healthy and thriving and presented with frequent regurgitation were not prescribed acid suppression medication at the first presentation. | 0 - 11 months | 14 | 12 | 24 | 14 | Consensus-based recommendation | Treatment | Overuse |
| GORD20 | Children with Barrett's Oesophagus had multiple biopsies obtained at time of endoscopy. | 0 - 15 years | 1 | 0 | 1 | 0 | Consensus-based recommendation | Treatment | Underuse |
| GORD21 | Children with Barrett's Oesophagus were prescribed acid suppression. | 0 - 15 years | 1 | 0 | 1 | 0 | Consensus-based recommendation | Treatment | Underuse |
| GORD22 | Older children/adolescents who presented with heartburn were assessed for lifestyle factors (diet, alcohol, weight, sleeping position, smoking). | 13 - 15 years | 3 | 0 | 3 | 1 | Grade A | Treatment | Underuse |
| GORD23 | Older children/adolescents who presented with heartburn were prescribed a PPI for 4 weeks. | 13 - 15 years | 4 | 0 | 2 | 0 | Grade A | Ongoing management | Underuse |
| GORD24 | Older children/adolescents who presented with heartburn, had been prescribed and used a PPI for 4 weeks, and their symptoms had resolved/improved were reviewed by their GP and had their PPI continued for 3 months. | 13 - 15 years | 2 | NA | NA | NA | Grade D | Ongoing management | Underuse |
| GORD25 | Older children/adolescents who presented with heartburn, had been prescribed and used a PPI for 4 weeks, and they had recurrent/persistent symptoms were reviewed by their GP and referred to a gastroenterologist. | 13 - 15 years | 1 | NA | NA | NA | Grade D | Ongoing management | Underuse |
| GORD26 | Infants/children (aged less than 18 months) with reflux oesophagitis had their family lifestyle factors recorded (diet, alcohol, weight, sleeping position, smoking). | 0 - 17 months | 6 | 2 | 13 | 7 | Grade A | Ongoing management | Underuse |
| GORD27 | Infants/children (aged less than 18 months) with reflux oesophagitis had their symptoms reassessed at each review. | 0 - 17 months | 5 | 1 | 8 | 3 | Grade A | Ongoing management | Underuse |
| GORD28 | Infants/children who had the presence of warning signs (see definition^) were referred to a paediatric gastroenterologist. | 0 - 15 years | 8 | 3 | 13 | 10 | Grade A | Ongoing management | Underuse |
| GORD29 | Infants/children who had difficulty swallowing or a history of obstruction were referred to a paediatric gastroenterologist. | 0 - 15 years | 1 | 3 | 7 | 4 | Consensus-based recommendation | Ongoing management | Underuse |
| GORD30 | Infants/children who had weight loss/anorexia/poor feeding were referred to a paediatric gastroenterologist. | 0 - 15 years | 6 | 3 | 14 | 11 | Consensus-based recommendation | Ongoing management | Underuse |
| GORD31 | Infants/children whose symptoms persisted during and after PPI therapy were referred to a paediatric gastroenterologist. | 0 - 15 years | 8 | 4 | 6 | 10 | Consensus-based recommendation | Ongoing management | Underuse |
| GORD32 | Infants/children with uncomplicated recurrent regurgitation who presented with projectile vomiting OR haematemesis OR bile-stained vomiting, were immediately referred to a hospital emergency department. | 0 - 15 years | 0 | 0 | NA | NA | Consensus-based recommendation | Ongoing management | Underuse |

**Legend**: ID=Identifier; GP=General Practitioner; P=Pediatrician; ED=Emergency Department; INPT=Inpatient; MC&S=Microscopy, Culture and Sensitivities; PPI=Proton-Pump Inhibitor.

^#^ Strength of recommendation as reported in individual CPGs. CPGs used a variety of classification schemes for allocating strength of recommendation in Grades (with A indicating the strongest recommendation in all classification schemes). Where Strength of Recommendation, or Level of Evidence, were not specified in the CPG, the term “Consensus-based recommendation” was assigned.

* The type of quality of care assessed was classified as underuse or overuse: underuse refers to actions which are recommended, but not undertaken; overuse refers to actions which are not indicated, or are contraindicated, in the context of the indicator’s inclusion criteria.

^^^ Includes: Bilious vomiting; Gastrointestinal bleeding; Hematemesis; Hematochezia; Consistently forceful vomiting; Onset of vomiting after 6 months of life; Failure to thrive; Diarrhea; Constipation; Fever; Lethargy; Hepatosplenomegaly; Bulging fontanelle; Macro/microcephaly; Seizures; Abdominal tenderness or distension; Documented or suspected genetic/metabolic syndrome.
