# Supplementary material for: Assessing the appropriateness of the management of gastro-oesophageal reflux in Australian children: a population-based sample survey
Source: Sci Rep. 2021 Apr 8;11:7744. doi: 10.1038/s41598-021-87369-7 (PMC8032666; doi:10.1038/s41598-021-87369-7)
Supplement: Supplementary file 2 — Supplementary Appendix 2. [file 41598_2021_87369_MOESM2_ESM.docx]

**Assessing the appropriateness of the management of Gastro-Oesophageal Reflux in Australian children: a population-based sample survey**

**Authors:**

Gaston Arnolda, PhD^1*^, Harriet Hiscock, MD^2,3,4^, David Moore, MBBS^5^, Glen Farrow, MBA^6^, Peter D. Hibbert, Grad Dip. Econ^1,7^, Louise K. Wiles, PhD^1,7^, Hseun P. Ting, MSc^1^, Charlotte J. Molloy, BBehSc^1,7^, Meagan Warwick, MSc^1^, and Jeffrey Braithwaite, PhD^1^, *on behalf of the CareTrack Kids Investigative Team*

^1^ Australian Institute of Health Innovation, Macquarie University, Level 6, 75 Talavera Road, Sydney, NSW 2109

^2^ Population Health Theme, Murdoch Children’s Research Institute, Royal Children’s Hospital, Flemington Road, Parkville, VIC 3052

^3^ Department of Paediatrics, The University of Melbourne, Melbourne, VIC 3010

^4^ School of Population and Global Health, The University of Melbourne, Melbourne, VIC 3010

^5^ Women’s and Children’s Hospital, North Adelaide, 72 King William Road, SA 5006

^6^ Sydney Children's Hospital Network, Prince of Wales Hospital, High Street, Randwick, NSW 2031

^7^ Centre for Population Health Research, School of Health Sciences, University of South Australia, 101 Currie Street, Adelaide, SA 5001

***Corresponding author:**

**Dr Gaston Arnolda**

Centre for Healthcare Resilience and Implementation Science

Australian Institute of Health Innovation, Macquarie University

Level 6, 75 Talavera Road, Macquarie University | NSW | 2109

**Email: gaston.arnolda@mq.edu.au**; Phone +61 2 9850 2411; Fax +61 2 9850 2499

# Appendix 2: Additional details relating to study methods

The report of top-level CareTrack Kids (CTK) paper[1] and its associated online appendix detail the methods of the larger study, which generated the data reported in this paper. Selected methods specifically relevant to GOR and GORD are described below.

**Sample size**

A visit was defined as an occasion of care: for inpatient care, an occasion of admitted care; for Emergency Department (ED) care, a single presentation; and for General Practitioners (GPs) or Paediatrician care, a consultation). Without adjustment for the design effect, a minimum of 400 visits per condition was required to obtain national estimates with 95% Confidence Interval (CI) and precision of +/- 5% at condition level. It was anticipated that loss of precision due to design effects would be largely offset by multiple eligible indicators per visit and additional visits identified by the secondary sampling (multiple visits for care of GOR/GORD for each medical record identified for sampling of GOR/GORD, and visits for care of GOR/GORD incidentally found in medical records identified for sampling other conditions).

**Sampling** **Process**

A multistage stratified random sampling process was implemented. For logistical efficiency, sampling targeted three states, Queensland (QLD), New South Wales (NSW) and South Australia (SA), in which 60.0% of the Australian population aged 15 years or younger resided in the 2012 and 2013 calendar years. All six paediatric tertiary hospitals (two in QLD, three in NSW, and one in SA) were targeted as they have state-wide coverage. State Departments of Health organize care within administrative units (‘health districts’): Hospital Health Services in QLD, Local Health Districts in NSW, and Local Health Networks in SA. For QLD, five health districts were targeted (two metropolitan, three regional), in NSW four (two metropolitan, two regional), and in SA three (two metropolitan, one regional).

**Recruitment of health care providers**

Within the selected health districts, the project approached all public hospitals, or private hospitals providing public services under contract, that had patient volumes of ≥2,000 paediatric ED presentations and ≥500 paediatric separations per year. The research team separately advertised the study to general practices and paediatricians and approached all the providers identified through internet searches, and via personal contacts. Within the selected sites, medical records were sampled for each condition targeted in that setting.

Recruitment of GPs and paediatricians was decentralized. Administrative details for refusal rates, from cold-calling or direct contact by clinicians who facilitated recruitment of their peers, were maintained on project laptops. At the end of recruitment all computers were decommissioned and cleaned, with the files archived on a USB drive. Unfortunately, the USB drives created during laptop decommissioning were misplaced and have not been able to be located. This did not affect the indicator adherence data, as the database was remotely located and updated regularly via the internet. Recruitment rates have therefore been estimated from recruitment spreadsheets emailed to administrative staff.

For GPs, emailed spreadsheets with late stage records could only be located for one state, South Australia. Based on this spreadsheet, 114 GPs were approached and 27 recruited, giving a recruitment rate of 23.7%; an additional GP, not listed on the available spreadsheet, was recruited subsequently and was not added to either the numerator or the denominator, for this estimate. The spreadsheet did not have clear information on eligibility, so it is likely that an unknown number of the 114 approached were ineligible because: 1) they were not open during the whole 2012-2013 survey period; 2) they saw no or few children; or 3) they were not confident in their ability to generate full listings of children with the target conditions, or they did not use one of the four practice software systems our surveyors were trained to search. The 23.7% recruitment rate is therefore likely to be an underestimate of the actual rate.

For paediatricians, emailed records with late stage records were located for all three states. Based on these spreadsheets, 80 eligible paediatricians were approached and 20 recruited, giving a recruitment rate of 25.0%.

Self-selection of GPs and paediatricians, and the estimated 24-25% recruitment rate, could lead to bias in the estimated rate of guideline adherence, arising from self-selection. It is plausible that self-selected practices were more confident of their guideline adherence, potentially leading to overestimation of the quality of care in the CTK study.

**Allocation of surveys to sampling units**

The number of GOR/GORD records targeted at each site was determined by a nominal allocation of the 400 records targeted, informed by data available at the time, supplemented by expert opinion, with planned over-sampling of settings where fewer occasions of care were expected.[1, 2] For hospitals, a fixed number was targeted at each site; for paediatricians, a fixed number was targeted initially, but this was abandoned as it was not possible to systematically identify patients by condition; for GPs, different combinations of conditions were targeted at each site, to reduce the workload associated with sampling.

**Data collection**

Nine experienced paediatric nurses were employed across the three states, with all nine assessing occasions of care for GOR/GORD. The surveyors undertook a one-week training program, prior to data collection. A surveyor’s manual was developed which included instructions, condition-specific definitions, inclusion and exclusion criteria, and guidance for assessing eligibility of each encounter for relevant indicators. Mock records were assessed during the surveying task for 6 of the 9 surveyors (2 had already terminated employment and 1 was excluded as their assessments may not have been made independently) and their results compared. A good level of agreement was found; κ = 0.76 (95%CI, 0.75-0.77; n = 1895) for the child’s eligibility for indicator assessment, and κ = 0.71 (95% CI, 0.69-0.73; n = 1009) for indicator assessment.[1]

A web-based tool, originally developed for the CareTrack Adults study,[3, 4] was designed to enter data during medical record review. Algorithms to filter indicators by setting, by age, or both, were embedded in the tool. Two indicators (GORD24-25) were restricted to GPs, one (GORD32) was restricted to GPs and paediatricians, and 20 were restricted by age criteria, as shown in eTable 1, Appendix 1.

Surveyors undertook criterion-based medical record reviews using the data collection tool. Surveyors assessed the record for evidence that the participant presented for management of GORD in the years 2012 and 2013. The surveyors responded to each indicator as ‘Yes’ (care provided during the encounter was consistent with the indicator), ‘No’, or ‘Not Applicable’ (NA; the indicator was not eligible for assessment). For example, a surveyor assessing an ED presentation for management of an infant with GORD, but without Barrett’s Oesophagus, would record ‘NA’ to indicators GORD20-GORD21 which are for infants with a diagnosis of Barrett’s Oesophagus.

**Analysis**

Survey or register-derived data were used to estimate the proportion of occasions of care for GORD.[5-12] The number of occasions of healthcare for each condition was thereby estimated for each site, and sampling weights were calculated using the methods detailed in eAppendix 4 of the report of the top-line CTK results (this Appendix can be accessed by request via the corresponding author, if required).[1]

State and healthcare setting were specified as strata, and the primary sampling unit (health district) was specified as the clustering unit. Pseudo-strata were constructed as necessary whenever there was only one cluster within a stratum, to avoid underestimation of the variance: specifically, GP and paediatrician data were collapsed to form a non-hospital pseudo-stratum in SA. Domain analysis was used for analysing indicators.[13, 14]

## References:

1. Braithwaite J, Hibbert PD, Jaffe A, et al. Quality of Health Care for Children in Australia, 2012-2013. *JAMA.* 2018;319:1113-24.

2. Hooper TD, Hibbert PD, Mealing N, et al. CareTrack Kids-part 2. Assessing the appropriateness of the healthcare delivered to Australian children: study protocol for a retrospective medical record review. *BMJ Open.* 2015;5:e007749.

3. Hunt TD, Ramanathan SA, Hannaford NA, et al. CareTrack Australia: assessing the appropriateness of adult healthcare: protocol for a retrospective medical record review. *BMJ Open.* 2012;2:e000665.

4. Runciman WB, Hunt TD, Hannaford NA, et al. CareTrack: assessing the appropriateness of health care delivery in Australia. *Med J Aust.* 2012;197:100-5.

5. Britt H, Miller GC, Henderson J, et al. (2013) General Practice Activity in Australia 2012-13: BEACH: Bettering the Evaluation and Care of Health. Sydney, AU: Sydney University Press.

6. Harrison C. BEACH 2012-13 weighted data on frequency of management of selected conditions, for children aged 0-15, by General Practitioners. [Personal communication]. Sydney, AU: Menzies Centre for Health Policy, School of Public Health, The University of Sydney; 2017.

7. Hiscock H, Danchin MH, Efron D, et al. Trends in paediatric practice in Australia: 2008 and 2013 national audits from the Australian Paediatric Research Network. *J Paediatr Child Health.* 2017;53:55-61.

8. Hiscock H. CAP 2013 data on frequency of management of selected conditions, for children aged 0-15, by paediatricians. [Personal communication]. Australian Paediatric Research Network; 2017.

9. Australian Institute of Health and Welfare. (2013) Australian hospital statistics 2012–13: Emergency Department care. Canberra, AU: AIHW.

10. Queensland Health, New South Wales Health, South Australian Department of Health. Emergency Department data on frequency of management of selected conditions, for children aged 0-15. [Personal communication]. 2017.

11. Australian Institute of Health and Welfare. (2014) Australian hospital statistics 2012–13. Canberra, AU: AIHW.

12. Australian Institute of Health and Welfare (2017) Inpatient separations for selected conditions, as identified by ICD-10 principal diagnoses. Canberra, AU: AIHW.

13. Lohr S. (2009) Sampling: design and analysis. Second ed. Boston, MA: Brooks/Cole.

14. Heeringa SG, West BT, Berglund PA. (2010) Applied survey data analysis. Boca Raton, FL: CRC Press.
